# Trends in the Prevalence, Awareness, Treatment, and Control of Hypertension in Nepal between 2000 and 2025: A Systematic Review and Meta-Analysis

**DOI:** 10.1155/2021/6610649

**Published:** 2021-03-02

**Authors:** Raja Ram Dhungana, Achyut Raj Pandey, Nipun Shrestha

**Affiliations:** ^1^Institute for Health and Sport, Victoria University, Melbourne, Australia; ^2^Nepal Family Development Foundation, Kathmandu, Nepal; ^3^Abt Associates, Kathmandu, Nepal

## Abstract

**Background:**

Understanding the burden and trend of hypertension and the associated care cascade can provide direction to the development of interventions preventing and controlling hypertension. This study aimed to assess prevalence and trends of hypertension and its awareness, treatment, and control in Nepal.

**Methods:**

We systematically searched CINAHL, Embase, ProQuest, PubMed, Web of Science, WorldCat, and government and health agency-owned websites to identify studies reporting prevalence of hypertension, awareness, treatment, and control in Nepal between 2000 and 2020. We applied the random-effects model to compute the pooled prevalence in the overall population and among subgroups in each 5-year interval period between 2000 and 2020. We used linear meta-regression analysis to predict hypertension from 2000 to 2025.

**Results:**

We identified 23 studies having a total of 84,006 participants. The pooled prevalence of hypertension, awareness, treatment, and control for 2016–2020 was 32% (95% CI: 23–40%), 50% (95% CI: 30–69%), 27% (95% CI: 19–34%), and 38% (95% CI: 28–48%), respectively. The prevalence of hypertension varied by age, gender, education, and geographical area. Hypertension increased by 6 percentage points (pp), awareness increased by 12 pp, treatment increased by 11 pp, and control increased by 3 pp over the 20 years studied. Since 2000, the rate of increment of hypertension has been 3.5 pp per decade, where 44.7% of men are expected to suffer from hypertension by 2025.

**Conclusion:**

The markedly increased prevalence of hypertension and relatively poor progress in hypertension awareness, treatment, and control in Nepal suggest that there is a need for hypertension preventive approaches as well as strategies to optimize hypertension care cascade.

## 1. Background

Hypertension is a major public health problem right around the world. Globally, it affects around 22% of the population aged 18 years and over and is responsible for an estimated 9.4 million deaths per year [1, 2]. It is the most common cause of cardiovascular diseases (CVDs) including coronary artery disease, congestive heart failure, renal insufficiency, peripheral vascular disease, and stroke [3–5]. Furthermore, it is the leading cause of CVD deaths. Indeed, 45% of deaths due to heart disease and 51% of deaths due to stroke were attributable to hypertension [1].

Approximately 75% of the world's hypertensive population live in low-income and middle-income countries (LMICs) [6]. Southeast Asia (25.1%) has the third-highest prevalence of hypertension after Africa (27.4%) and the Eastern Mediterranean (26.3%) [2]. Based on the World Health Organization (WHO) estimation for 2015, Nepal was ranked third in the prevalence of hypertension (29.4%) in South Asia, following Afghanistan (30.6%) and Pakistan (30.5%) [2].

The increasing prevalence of hypertension in LMICs is a major concern. The prevalence of hypertension in LMICs increased by 7.7% between 2000 and 2010 [6]. Evidence suggests that the burden of hypertension is also rising in Nepal. A study conducted in the rural areas of Kathmandu District reported a three-fold increase in the prevalence of hypertension prevalence in 25 years [7]. The last two periodic surveys conducted in Nepal (STEPS survey 2007 and 2019) showed that the prevalence of hypertension had increased from 21.5% [8] to 24.5% in Nepal [9]. Other studies reported largely varied findings on hypertension, with prevalence ranging from 19.6% [10] to 25.7% [9] at the national level; 15.1% [11] to 38.9% [12] at the regional level; 21.7% [11] to 48.1% [12] in males; 10.5% [11] to 35.2% [12] in females; 22.5% [13] to 38.6% [14] in rural areas; and 32.5% [15] to 38.9% [12] in urban areas. Huang et al. [16]conducted a meta-analysis to pool the prevalence of hypertension in Nepal in 2018. Their study, however, failed to accommodate the findings of the STEPS surveys of 2003, 2005, 2007, and 2019 [17–20] and limited the analysis to estimating the prevalence of hypertension. Additionally, no studies have attempted to systematically review the literature on the awareness, treatment, and control of hypertension in Nepal.

To fill this gap and thereby determine the actual prevalence and distribution of hypertension as well as its trend over the years in Nepal, it is essential to systematically synthesize all available literature on hypertension prevalence in Nepal. Understanding trends in and patterns of hypertension and its cascade of care—awareness, treatment, and control—will make a significant contribution toward the prevention and effective management of high blood pressure in Nepal. This study aimed to estimate the prevalence, awareness, treatment, and control of hypertension; depict the trends in these rates over the last 20 years, between 2000 and 2020; and project their rates for 2025.

## 2. Methods

We used systematic review and meta-analysis following the PRISMA guidelines [21] and reported the findings using the PRISMA checklist. We defined the eligibility criteria of the studies based on population, outcome of interest, and study design.

### 2.1. Search Strategy

We systematically searched six databases: PubMed, Embase, Web of Science, CINAHL through EBSCOHost, ProQuest, and WorldCat. In order to maximise the sensitivity of the search strategy, we used broad search keywords such as “hypertension” or “blood pressure” in combination with “Nepal” instead of applying specific words related to hypertension awareness, treatment, and control and searched in all text fields. We applied the same search strategy for *NepJOL, Journal of Nepal Health Research Council, Journal of Kathmandu Medical College*, and *Nepal Medical College Journal*. In addition, we ran an open search on the websites of Nepal's Ministry of Health and other national and international health agencies to locate reports not published in the journals. Finally, we conducted both a forward and a backward citation search of the included articles using Google Scholar and the reference list of the included articles, respectively.

The searched records were sent to EndNote X8 (Clarivate Analytics, Philadelphia, PA, USA) and Rayyan QCRI [22] to carry out screening and data extraction.

### 2.2. Study Selection

We included those studies which reported population-based estimates of the prevalence, awareness, treatment, and control of hypertension in Nepal. Hypertension was defined as the state of having systolic blood pressure ≥140 mmHg and/or diastolic blood pressure ≥190 mmHg and/or the use of antihypertensive medication. Hypertension awareness means knowing that one has high blood pressure as per the diagnosis of a doctor or health worker. Hypertension treatment is the use of any antihypertensive medication for lowering blood pressure. We defined hypertension control as the reduction of systolic and diastolic blood pressure readings to below 140 mmHg and 90 mmHg, respectively, among treated participants.

Authors RRD and ARP independently screened the titles, abstracts, and full texts of articles using predefined study selection criteria. Studies had to have had a cross-sectional design or survey, been conducted among populations 15 years and older between 2000 and 2020, and been published either in English or Nepali. We excluded studies that had sample sizes less than 400 and that were conducted in a specific population such as children and diabetic patients or in specific settings such as a hospital. Studies that applied nonprobability sampling methods were also not eligible for our study. If more than one article was published using the same data, we selected the study that contained more detailed information on the parameters of interest. The screening was conducted independently by RRD and ARP and all disagreements in the selection of studies were settled by consensus between the two authors.

### 2.3. Data Extraction

We extracted the required data including the major characteristics of the selected studies, particularly those related to study design, study types, study participants, study setting, year of the study commencement, sample size, number of events, and information to assess the risk of bias. When certain required data were not reported in the studies, we either contacted the primary authors or used the original data to extract the information ourselves.

### 2.4. Quality Assessment

We used the JBI checklist to assess the methodological quality of the selected studies [23]. This e-tool has checklists of nine items. Each item offers “yes,” “no,” “unclear,” and “not applicable” as the four options for response. For each “yes” response, the given study was assigned 1 point. A study could, therefore, get an overall score between zero and nine points. For this review, we used the following scoring system for overall methodological quality: <6 points = “low”; 6–7 points = “fair”; 8–9 points = “good.” The two authors independently checked the quality of the studies. To resolve discrepancies between the assessment scores of the two authors (RRD and ARP), a third author (NS) also assessed the quality of the studies using the same tools. All conflicts were resolved by consensus.

### 2.5. Data Analysis

We analysed the data using STATA software version 16.0 (Stata Corporation, College Station, TX, USA). The extracted data on sample size and events were used to calculate prevalence estimates. To estimate the pooled effect size, we chose the random-effects model and used the I-squared index (I-squared ≥50% indicating heterogeneity) and Cochrane *Q* test to determine the extent of heterogeneity. We created five-year interval period—2000–2005, 2006–2010, 2011–2015, and 2016–2020—and calculated the weighted prevalence of hypertension, awareness, treatment, and control for each period. These five-year intervals matched the five-year strategic health planning process in Nepal including the Nepal Health Sector Programme (NHSP) I, NHSP II, and NHSP III and the Nepal Health Sector Strategy. The time interval was also built around the periodic STEP-wise approach to the risk-factor surveillance of chronic disease (STEPS surveys) conducted in Nepal at five-year intervals. We also performed subgroup analysis by gender, age, education, study setting (rural vs urban), and provinces. In addition, we fitted linear and nonlinear meta-regression models and predicted the prevalence of hypertension, awareness, treatment, and control for 2020 to 2025. For the linear model, we assumed that the proportion of hypertension would continue to increase or decrease at the annual rate, where the beta coefficient represented the change in proportion over time. For nonlinear meta-regression analysis, we transformed the independent variable “survey years” (centred at 2001) into the square and cubic forms and included it in the existing linear models. The fit of the model was compared using R-square, F values, and chi-square statistics. The predicted prevalence was also plotted on a line graph to depict the gap between the existing trend in hypertension and the global target of a 25% reduction in hypertension by 2025. For sensitivity analysis, the models were also fitted separately for the studies conducted nationwide and compared with the main models.

We constructed the symmetry of funnel plots and performed Egger's regression asymmetry test to evaluate publication bias. All the estimates are presented in 95% confidence interval (CI). A *p* value less than 0.05 was considered statistically significant.

## 3. Results

**Figure 1:**
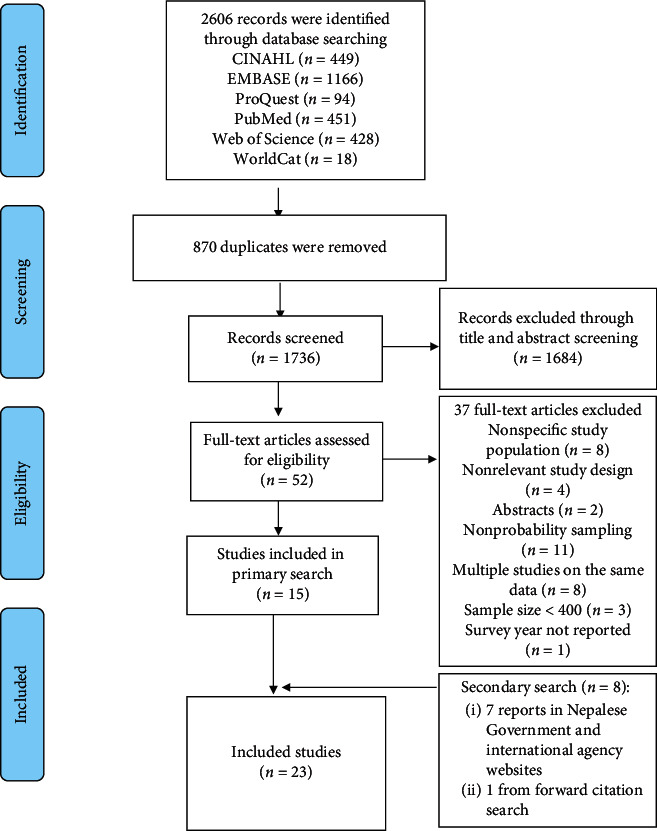
Study selection flow diagram.

In total, 2606 records were retrieved from the systematic search of the six electronic databases. After removing duplicates and nonrelevant articles from the title and abstract screening, 52 full-text articles were assessed for eligibility. Of them, 15 studies [7, 12, 15, 24–35] met the inclusion criteria. We then used the raw data from four of those eligible studies to reproduce results for our purpose instead of extracting information from the published article [12, 15, 29, 34]. Out of 37 excluded articles, 11 studies, including May Measurement Month Campaigns, applied convenient or other nonprobability sampling methods to select the participants [22, 36–44]. In addition, we identified seven reports published on government and international agency websites [17–20, 45–47]. Three of the reports had publicly available data [18, 45, 47], which we utilized to generate and extract the required information for this study. One additional study [48] was located using the forward citation method. In the end, we included 23 studies for our analysis (Figure 1).

### 3.1. Study Characteristics

The total participants from the 23 selected studies were 84,006, and the sample sizes of individual studies ranged from 406 to 15,934. The majority of the participants were between 20 to 70 years of age and were females. Two studies, those conducted by Vaidya et al. and Khan et al., studied either only male or only female participants. Eight out of 23 studies were from Province 3 (Bagmati), and five studies including periodic STEPS surveys and the Demographic Health Survey (DHS) were nationally representative studies. Half of the studies (12/23) were conducted in urban settings. Six studies were conducted between 2000 and 2005, four studies between 2006 and 2010, eight studies between 2011 and 2015, and the remaining five studies were completed between 2016 and 2020 (Table 1).

Fifteen studies reported the proportion of hypertension awareness among hypertensive participants. Likewise, 16 studies also assessed hypertension treatment and 12 studies presented findings on hypertension control among the hypertensive participants who were being treated.

Sixteen studies reported the response rate, which ranged from 85% to 99%. In the quality assessment of the studies, 11 studies [12, 15, 19, 20, 26, 27, 29, 34, 45–47] were categorized as good quality studies (JBI checklist = 8-9) and 12 studies [7, 17, 18, 24, 25, 28, 30–33, 35, 48] were fair in quality (JBI checklist = 6-7). Details of the quality assessment of studies are available in Supplementary file 1.

### 3.2. Prevalence, Awareness, Treatment, and Control of Hypertension between 2000 and 2020

The pooled prevalence of hypertension during the five-year intervals 2000–2005, 2006–2010, and 2016–2020 was 26%, 29%, and 32% respectively, suggesting a 6 pp increment over the last 20 years (Table 2).

The prevalence of hypertension awareness, treatment, and control in the period 2016–2020 was 50%, 27%, and 38%, respectively, figures which represented increases of 12%, 11%, and 3% compared to the estimates from the period 2000–2005. The hypertension control rate among total hypertensive participants during that same time period was 8% (95% CI: 7–12%).

### 3.3. Prevalence of Hypertension, Awareness, Treatment, and Control by Subgroups

**Figure 2:**
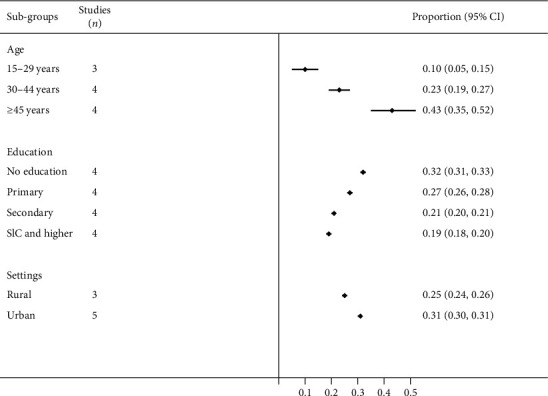
Prevalence of hypertension by subgroups in the period 2016–2020.

Between the periods 2000–2005 and 2016–2020, the prevalence of hypertension increased from 25% to 38% in men and 21% to 28% in women (Table 2). During the same time period, the prevalence of hypertension increased from 19% to 33% in Province 3 (Bagmati) and 26% to 33% in urban areas. Details of prevalence estimates by subgroups between 2000–2020 are available in Supplementary file 2. In the period 2016–2020, the pooled prevalence of hypertension varied largely by subgroups and was highest among men, the elderly, those with no or little education, and those who resided in urban settings (Figure 2).

**Figure 3:**
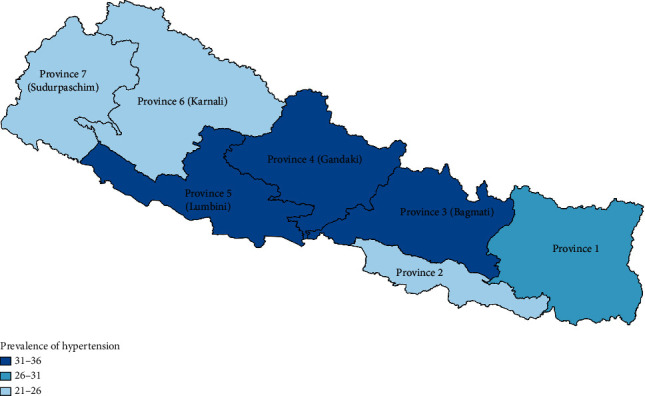
Prevalence of hypertension by province in the period 2016–2020.

In terms of regional distribution, the highest and the lowest estimates of the prevalence of hypertension were observed in Province 4 (36%, 95% CI: 23–48%) and Province 7 (21%, 95% CI: 13–29%) in the period 2016–2020 (Figure 3).

In contrast to hypertension prevalence, the pooled estimates of hypertension awareness, treatment, and control were higher in women than men (Supplementary file 2).

### 3.4. Trends in and Projections of Hypertension, Awareness, Treatment, and Control

None of the quadratic and cubic models in meta-regression analysis were significant and their R-squared statistics were lower than those for the linear models (Supplementary file 3). In linear meta-regression analysis, the prevalence of hypertension in men (B = 0.009, 95% CI: 0.0023–0.016) and hypertension treatment (B = 0.0098, 95% CI: 0.00002–0.0196) were significantly associated with the survey year. The prevalence of hypertension in men would, as the model predicted, increase by 1.0 pp annually and reach 44.7% by 2025. Similarly, the hypertension treatment rate would reach 37.2% by 2025 (Table 3).

**Figure 4:**
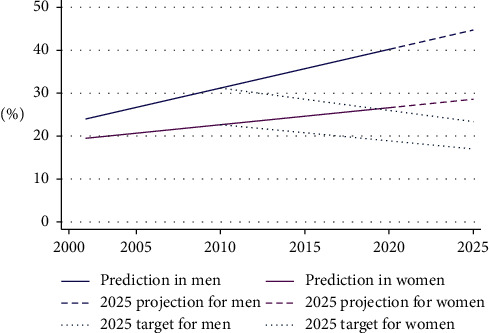
Prediction of hypertension and a 25% reduction target by 2025.

The predictions for hypertension in men and women for 2010 were 31% and 22.7%, respectively. Based on the global target of a 25% reduction in hypertension by 2025, that rate should be reduced to 23.4% in men and 17% in women.  Figure 4 depicts the gap in the target set for a 25% reduction by 2025 and the projected prevalence.

### 3.5. Sensitivity Analysis and Publication Bias

No significant difference in prevalence estimates was observed between studies of different qualities (B =−0.0155, 0 95% CI: 0−0.086–0.055) or between nationwide and regional studies (B = 0.001, 0 95% CI = −0.078–0.081). The meta-regression analysis conducted only among nationwide studies showed no significant association between hypertension prevalence and survey year. The symmetric distribution of the studies in a funnel plot (Supplementary file 4) and results from Egger's test (*p*=0.5) showed no substantial publication bias.

## 4. Discussion

This study synthesized the evidence on the prevalence, awareness, treatment, and control of hypertension in Nepal from 2000 to 2020 and found that, in the period 2016–2020, 32% of Nepali were hypertensives, 50% of them were aware of their hypertensive status, 27% were under medication, and 38% of the treated hypertensives had their blood pressure under control. The findings suggested that the burden of hypertension is highest among men, older people, people with no or low educational attainment, and those who live in urban areas and Province 4 (Gandaki). Prevalence estimated at five-year intervals suggested that there had been an upward trend in the burden of hypertension in Nepal but relatively small progress in hypertension control over the last two decades.

Our finding on the prevalence of hypertension (32%) for the period 2016–2020 is higher than the prevalence reported by Young et al. (27%), WHO estimates for Nepal (29.4%), and the estimates for India (25.8%), Bangladesh (24.7%), low-income countries (23.1%) [49], and the world as a whole (the pooled prevalence of 200 countries) [50].

This study showed that the burden of hypertension, particularly in men, has increased significantly in the last 20 years. If the current linear trend holds, the prevalence of high blood pressure in men is projected to be 44.7% by 2025. The global trend in the burden of hypertension, however, suggests otherwise. A recent study that analysed data from 19.1 million participants found that the age-standardized prevalence of high blood pressure had decreased from 29.5% to 24.1% in men and 26.1% to 20.1% in women between 1975 and 2015 [50]. Despite the rapid decline in hypertension in high-income countries, it remained unchanged or is still increasing in South Asia [50]. A regional study from India reported a 6.6 pp increase (vs 6% in our study) in raised blood pressure in the nation's selected urban population from 1991 to 2015 [51]. A meta-analysis from Bangladesh predicted a 5 pp increase in hypertension in each decade [52]. Subgroup variation in the prevalence of hypertension particularly by age, gender, education, and setting is consistent with other studies published both in Nepal and outside [16, 53]. Variations in the prevalence of hypertension across the provinces could be related to the differences in dietary habits and lifestyles associated with the population composition including ethnicity.

The findings on hypertension awareness (29%), treatment (19%), and control (9% of all hypertensives) in the period 2006–2010 were comparable to the rates documented in other LMICs in 2010, where, of all hypertensives, 37.9% were aware, 29.0% were treated, and 7.7% had controlled their hypertension [6]. However, our estimates were lower than those reported in other South Asian countries, namely, Bangladesh, India, and Pakistan, where 40.4% of hypertensives were aware, 31.9% were taking antihypertensive medication, and 12.9% had controlled their blood pressure in 2009 [54].

The 20-year trend in the prevalence of hypertension awareness, treatment, and control suggested a very insignificant improvement in hypertension cascade care in Nepal. The rates of awareness, treatment, and control increased by 12%, 11%, and 3%, respectively. Evidence shows that, mostly around 1990, a significant improvement in hypertension cascade care was observed in high-income countries, and that even today, countries like Canada, Germany, South Korea, and the USA maintain the highest rates of awareness, treatment, and control globally [55]. Those countries could increase the rate of hypertension control by promoting health education and effective hypertension care services [56]. For example, between 1992 and 2013, the Canadian Hypertension Education Program was linked to an increase in treatment rates from 35% to 80% and in control rates from 13% to 68% [57]. A lower level of awareness, treatment, and control in Nepal indicates that there is much scope for improvement in the hypertension cascade. Progress can be achieved through the universal roll-out of the Package of Essential Noncommunicable Diseases (PEN). The recent political restructuring moving the nation from a unitary to a federal structure with seven provincial and 753 local governments provides an additional opportunity of introducing locally contextualized interventions based on the characteristics of residents at the provincial and local levels.

The growing burden of hypertension and poor performance in hypertension care cascade could create enormous healthcare challenges in Nepal. Along with high morbidity and mortality, uncontrolled hypertension could cause up to 25% of all health expenditures [58]. In South Asia, the healthcare cost attributed to high blood pressure is expected to be USD 30657 million over a 10-year period [58]. Evidence suggests that nonpharmacological interventions such as smoking cessation, reduction in dietary sodium intake, physical exercise, weight reduction, and yoga, among others, can prevent and control hypertension [59]. These measures are cost-effective as well. For example, Webb et al. estimated that a 10% reduction in sodium intake within each country over a 10-year period could prevent approximately 5.8 million DALYs/year related to CVD and hypertension [60]. Nepal has already introduced policies related to tobacco [61] and alcohol [62], both of which are not specific, but can be linked to hypertension prevention and control. The country has yet to formulate policies specific to promoting physical activity and salt reduction in the population.

Studies suggest that the poor treatment and control of hypertension are associated with several factors relating to patients, providers, and healthcare services. These include patients' knowledge and practice [63, 64], providers' skills and motivation [63, 65, 66], and the availability and affordability of healthcare services [56, 63]. Interventions targeting providers, patients, or healthcare services and delivered at individual, community, health facility, or policy level such as home-based blood pressure monitoring [67], task shifting [68], team-based care [69], and health system financing [56] could be effective in controlling blood pressure. Among the many policy interventions in Nepal, only the PEN and the Multi-Sectoral Action Plan for the Prevention and Control of NCDs (2014–2020) are specific to hypertension and CVDs and aim to effectively manage major NCDs, including CVD, and reduce hypertension by 25% by 2025. However, our findings indicated that there is a huge gap between the projected estimate and the target for hypertension reduction by 2025.

This review has some limitations. Though the proportion of hypertension was available in all 23 included studies, some studies lacked age, sex, and education disaggregated data. The missing data could have affected the estimates generated from subgroup analysis. Likewise, information on hypertension awareness, treatment, and control was also not reported in several studies. As the number of hypertensive cases rather than the total sample was taken as a denominator for hypertension awareness, treatment, and control, the estimates had a wide confidence interval. That fact may suggest the need for a large population-based study designed to precisely estimate the parameters of the hypertension care cascade. Similarly, our assumption about the linearity of the trend may not hold true until 2025. Nonetheless, this is the first study to estimate the prevalence of the hypertension cascade of care and depict the trend in the burden of hypertension in Nepal. The study's findings will help policymakers and related stakeholders to evaluate current strategies and prioritize intervention designed to tackle the burgeoning burden of hypertension and improve its awareness, treatment, and control in Nepal.

## 5. Conclusions

The pooled estimates of the prevalence and the 20-year trends of hypertension suggest that the burden of hypertension is burgeoning in Nepal and is disproportionately affecting men, the elderly, those without formal education, and those who reside in urban areas or Province 4 (Gandaki). The low prevalence of and limited progress in the awareness, treatment, and control of hypertension in the last 20 years indicates poor performance in the cascade of care for hypertension. In order to achieve a 25% reduction in hypertension by 2025 and avoid the unavoidable health and economic consequences of uncontrolled hypertension, it is urgent to accelerate the implementation of interventions preventing and controlling hypertension in Nepal.

## Figures and Tables

**Table 1:** Study characteristics.

Sn.	Study ID	Survey year	Age range (in years)	Sample size (M/F)	HTN (M/F)	A/T/C	Setting	Study area	Quality
1	Shrestha et al.	2002	≥40	1012 (412/589)	236 (99/137)	(NA/NA/NA)	Urban	Multiples	Good
2	STEPS survey	2003	25–64	2030 (1010/1020)	382 (205/177)	(13/21/NA)	Urban	Province 3	Fair
3	Sharma et al.	205	≥20	3218 (1542/1676)	1243 (NA/NA)	659/336/136	Urban	Province 1	Fair
4	Vaidya et al.	2005	≥35	100 (1000/-)	227 (227/-)	NA/NA/117	Urban	Province 1	Fair
5	Sharma et al.	2005	18–97	1114 (541/573)	219 (120/99)	9/42/15	Urban	Province 3	Fair
6	STEPS survey	2005	15–64	7584 (3586/3998)	2199 (1210/989)	537/246/69	Both	Multiples	Fair
7	STEPS survey	2007	15–64	4328 (1907/2421)	1016 (526/490)	276/166/NA	Both	Nationwide	Good
8	Vaidya et al.	2006	≥21	1218 (527/691)	412 (202/210)	131/97/39	Rural	Province 3	Fair
9	Koju et al.	2007	18–88	796 (306/490)	230 (88/142)	69/42/NA	Urban	Province 3	Fair
10	Khan et al.	2008	16.4–71.2	15934 (-/15934)	530 (-/530)	NA/NA/NA	Rural	Province 2	Fair
11	Vaidya et al.	2011	25–59	777 (229/548)	168 (NA/NA)	NA/NA/NA	Urban	Province 3	Good
12	Neupane et al.	2013	25–65	2815 (971/1844)	838 (369/469)	390/257/128	Urban	Province 4	Good
13	Adhikari et al.	2013	15–64	1240 (665/575)	276 (165/111)	NA/NA/NA	Both	Multiples	Fair
14	STEPS survey	2013	15–69	4124 (1326/2798)	1209 (499/710)	286/164/49	Both	Nationwide	Good
15	Dhungana et al.	2014	20–50	406 (176/230)	50 (NA/NA)	NA/NA/NA	Rural	Province 3	Good
16	Aryalet al.	2015	≥30	521 (231/290)	198 (108/90)	NA/NA/NA	Both	Provinces 4 and 6	Fair
17	Dhungana et al.	2015	18–70	587 (242/345)	191 (93/98)	118/93/46	Urban	Province 3	Good
18	Karmacharya et al.	2015	≥18	1073 (446/627)	298 (167/131)	130/99/35	Urban	Province 3	Fair
19	Khanal et al.	2016	≥30	1159 (335/824)	451 (161/290)	241/131/37	Urban	Province 6	Good
20	Maharjan B	2016	20–59	580 (264/316)	215 (110/105)	135/100/NA	Urban	Province 3	Fair
21	DHS	2017	≥15	14351 (6153/8198)	2796 (1414/1382)	1599/537/272	Both	Nationwide	Good
22	NCDs survey	2018	≥20	12557 (4908/7649)	4504 (2131/2373)	NA/1291/429	Both	Nationwide	Good
23	STEPS survey	2019	15–69	5582 (1997/3585)	1575 (736/839)	398/205/78	Both	Nationwide	Good

Note: M, male; F, female; HTN, hypertension; NA, not available; A, awareness; T, treatment; C, control.

**Table 2:** Prevalence, awareness, treatment, and control of hypertension (2000–2020).

Parameters	Pooled estimate (95% CI)				
	2000–2005	2006–2010	2011–2015	2016–2020	Percent point change in 20 years
Hypertension	26% (19–33%)	29% (22–35%)	27% (22–31%)	32% (23–40%)	6%
Hypertension in men	25% (18–31%)	31% (25–38%)	37% (31–42%)	38% (28–49%)	13%
Hypertension in women	21% (16–25%)	21% (7–34%)	25% (22–27%)	28% (20–35%)	7%
Hypertension awareness	38% (21–54%)	29% (26–32%)	44% (28–60%)	50% (30–69%)	12%
Hypertension treatment	16% (7–25%)	19% (15–24%)	31% (17–45%)	27% (19–34%)	11%
Hypertension control (in all hypertensives)	7% (1–13%)	9% (7–13%)	13% (6–21%)	8% (6–11%)	1%
Hypertension control (in treated hypertensives)	35% (25–44%)	40% (31–50%)	41% (30–52%)	38% (28–48%)	3%

**Table 3:** Projected prevalence of hypertension, awareness, treatment, and control for 2025.

	Annual increase rate (pp)	Projected prevalence in 2025 (95% CI)
Hypertension	0.35	32.5% (23.5–41.4%)
Hypertension (men)	1.0^∗∗^	44.7% (34.7–54.7%)
Hypertension (women)	0.4	28.6% (19.0–38.2%)
Hypertension awareness	1.1	55.9% (36.5–75.4%)
Hypertension treatment	1.0^*∗*^	37.2% (22.8–51.6%)
Hypertension control (in all hypertensives)	0.2	11.7% (3.4–19.4%)
Hypertension control (in treated hypertensives)	0.3	41.4% (28.5–54.4%)

Note: ^*∗*^*p* < 0.05; ^*∗∗*^*p*<0.01.

## Data Availability

All data generated or analysed during this study are included in this published article and its supplementary materials.

## Supplementary Materials

Supplementary Materials Supplementary file 1. Quality assessment (xlsx 13 KB): the Joanna Briggs Institute Critical Appraisal tools (JBI) were used to assess the methodological quality of the selected studies. The tool has nine item checklists for different types of study design, with “Yes,” “No,” and “Unexplained”' response options. Supplementary file 2. Prevalence, awareness, treatment, and control of hypertension by subgroups (xlsx 14 KB): prevalence, awareness, treatment, and control of hypertension were estimated based on different age groups, gender, education, and geographical variations for 2000–2005, 2006–2010, 2011–2015, and 2016–2020. Supplementary file 3. Meta-regression models (PDF 114 KB): linear, quadratic, and cubic models were fitted for assessing the trend of prevalence, awareness, treatment, and control of hypertension between 2000–2025 and predicted values were plotted against the survey year in line graphs. Supplementary file 4. Funnel plot (PDF 22 KB): a funnel plot was constructed to assess the symmetry of the distribution of the included studies.
