# Supplementary material for: Trends in the Prevalence, Awareness, Treatment, and Control of Hypertension in Nepal between 2000 and 2025: A Systematic Review and Meta-Analysis
Source: Int J Hypertens. 2021 Mar 2;2021:6610649. doi: 10.1155/2021/6610649 (PMC7952181; doi:10.1155/2021/6610649)
Supplement: Supplementary Materials — Supplementary file 1. Quality assessment (xlsx 13 KB): the Joanna Briggs Institute Critical Appraisal tools (JBI) were used to assess the methodological quality of the selected studies. The tool has nine item checklists for different types of study design, with “Yes,” “No,” and “Unexplained”' response options. Supplementary file 2. Prevalence, awareness, treatment, and control of hypertension by subgroups (xlsx 14 KB): prevalence, awareness, treatment, and control of hypertension were estimated based on different age groups, gender, education, and geographical variations for 2000–2005, 2006–2010, 2011–2015, and 2016–2020. Supplementary file 3. Meta-regression models (PDF 114 KB): linear, quadratic, and cubic models were fitted for assessing the trend of prevalence, awareness, treatment, and control of hypertension between 2000–2025 and predicted values were plotted against the survey year in line graphs. Supplementary file 4. Funnel plot (PDF 22 KB): a funnel plot was constructed to assess the symmetry of the distribution of the included studies. [file 6610649.f1.zip › 6610649.f1/Supplementary file 1. Quality assessment.pdf]

Sn	Items	Study ID<br>Dhungana et al. (2014)	Vaidya et al. (2012)	Khan et al. (2013)
1	Was the sample frame appropriate to address the target population?	Y	y	N
2	Were study participants sampled in an appropriate way	Y	y	N
3	Was the sample size adequate	Y	y	Y
4	Were the study subjects and the setting described in detail?	Y	y	Y
5	Was the data analysis conducted with sufficient coverage of the identified sample?	Y	y	Y
6	Were valid methods used for the identification of the condition?	Y	Y	Y
7	Was the condition measured in a standard, reliable way for all participants?	Y	N	Y
8	Was there appropriate statistical analysis?	Y	y	Y
9	Was the response rate adequate, and if not, was the low response rate managed appropriately?	Y	N	N

Note: Y, Yes; N, No; U, Unclear; NA, Not applicable

Sharma et al. (2013)	Chataut et al. (2015)	Karmacharya et al. (2017)	Neupane et al. (2017)	Adhikari et al. (2014)	Koju et al. (2010)	Vaidya et al. (2007)	Sharma et al. (2006)	Shrestha et al. (2006)
Y	Y	U	Y	Y	Y	Y	Y	Y
U	U	Y	Y	Y	Y	Y	Y	Y
Y	Y	Y	Y	Y	Y	Y	Y	Y
Y	N	Y	Y	Y	Y	Y	Y	Y
Y	Y	Y	Y	Y	Y	Y	Y	Y
U	Y	Y	Y	U	Y	Y	Y	Y
U	N	Y	Y	U	N	N	N	Y
Y	Y	Y	Y	Y	Y	N	Y	Y
Y	U	U	Y	U	N	N	U	N

Dhungana et al. (2016)	Khanal et al. (2017)	Vaidya et al. (2013)	STEPs Survey 2019	STEPs Survey 2013	STEPs Survey 2007	STEPs Survey 2005	STEPs Survey 2003	DHS 2018	NCD survey 2018
Y	Y	Y	Y	Y	Y	Y	Y	Y	Y
Y	Y	Y	Y	Y	Y	Y	Y	Y	Y
Y	Y	Y	Y	Y	Y	Y	Y	Y	Y
Y	Y	Y	Y	Y	Y	Y	Y	Y	Y
Y	Y	Y	Y	Y	Y	Y	Y	Y	Y
Y	Y	Y	Y	Y	Y	Y	Y	Y	Y
Y	Y	Y	Y	Y	Y	N	N	Y	Y
Y	Y	Y	Y	Y	Y	U	U	Y	Y
Y	Y	N	Y	Y	U	U	U	Y	Y

Maharjan B	Aryal et al. (2019)
Y	U
U	Y
Y	Y
Y	Y
Y	Y
Y	Y
U	Y
Y	N
U	U
