# Supplementary figures and images for: Trends in the Prevalence, Awareness, Treatment, and Control of Hypertension in Nepal between 2000 and 2025: A Systematic Review and Meta-Analysis
Source: Int J Hypertens. 2021 Mar 2;2021:6610649. doi: 10.1155/2021/6610649 (PMC7952181; doi:10.1155/2021/6610649)

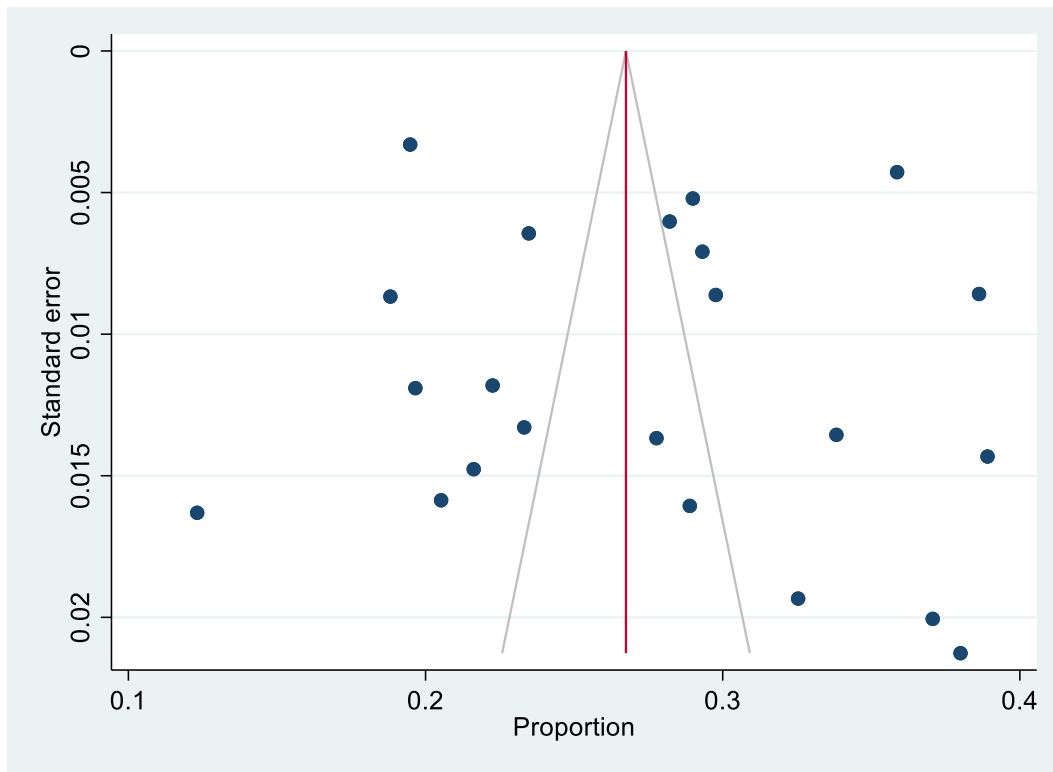

Fig 1. Funnel plots

Supplement: Supplementary Materials — Supplementary file 1. Quality assessment (xlsx 13 KB): the Joanna Briggs Institute Critical Appraisal tools (JBI) were used to assess the methodological quality of the selected studies. The tool has nine item checklists for different types of study design, with “Yes,” “No,” and “Unexplained”' response options. Supplementary file 2. Prevalence, awareness, treatment, and control of hypertension by subgroups (xlsx 14 KB): prevalence, awareness, treatment, and control of hypertension were estimated based on different age groups, gender, education, and geographical variations for 2000–2005, 2006–2010, 2011–2015, and 2016–2020. Supplementary file 3. Meta-regression models (PDF 114 KB): linear, quadratic, and cubic models were fitted for assessing the trend of prevalence, awareness, treatment, and control of hypertension between 2000–2025 and predicted values were plotted against the survey year in line graphs. Supplementary file 4. Funnel plot (PDF 22 KB): a funnel plot was constructed to assess the symmetry of the distribution of the included studies. [file 6610649.f1.zip › 6610649.f1/Supplementary file 4. Funnel plot.pdf]
